# Continuous perioperative heart rate variability monitoring in video-assisted thoracoscopic surgery lobectomy—a pilot study

**DOI:** 10.1007/s10877-023-01016-2

**Published:** 2023-05-27

**Authors:** Mikkel Nicklas Frandsen, Lin Huang, René Horsleben Petersen, Nicolai Bang Foss, Jesper Mehlsen, Henrik Kehlet

**Affiliations:** 1 Section for Surgical Pathophysiology, Copenhagen University Hospital, Rigshospitalet, Copenhagen, Denmark; 2 Department of Cardiothoracic Surgery, Copenhagen University Hospital, Rigshospitalet, Copenhagen, Denmark; 3 Department of Anesthesiology, Hvidovre University Hospital, Hvidovre, Denmark

**Keywords:** Heart rate variability, VATS, Enhanced recovery after surgery, Surgical risk stratification, Orthostatic intolerance, Postoperative atrial fibrillation

## Abstract

Heart rate variability (HRV) is a measure of cardiac autonomic modulation and is potentially related to hypotension, postoperative atrial fibrillation, and orthostatic intolerance. However, there is a lack of knowledge on which specific time points and indices to measure. To improve future study design, there is a need for procedure-specific studies in an enhanced recovery after surgery (ERAS) video-assisted thoracic surgery (VATS) lobectomy setting, and for continuous measurement of perioperative HRV. HRV was measured continuously from 2 days before until 9 days after VATS lobectomy in 28 patients. After VATS lobectomy, with median length of stay = 4 days, the standard deviation between normal-to-normal beats and the total power of HRV were reduced for 8 days during the night and day times, while low-to-high frequency variation and detrended fluctuation analysis were stable. This is the first detailed study to show that HRV measures of total variability were reduced following ERAS VATS lobectomy, while other measures were more stable. Further, preoperative HRV measures showed circadian variation. The patch was well tolerated among participants, but actions should be taken to ensure proper mounting of the measuring device. These results demonstrate a valid design platform for future HRV studies in relation to postoperative outcomes.

**Supplementary Information:**

The online version contains supplementary material available at 10.1007/s10877-023-01016-2.

## Introduction

Heart rate variability (HRV) measures derived from the ECG is associated with and a potential predictor of intra- and postoperative outcome [1, 2] and may be associated with postoperative atrial fibrillation following pulmonary lobectomy, in line with what has been found in coronary artery bypass surgery [1]. HRV has many differing and partly unknown physiological correlates and causes, reviewed elsewhere [3–5] but the questions of when and which parameters to measure in different surgical procedures have not been adequately addressed making it difficult to design and compare studies.

To better understand the time-course and pathophysiological impact of video-assisted thoracic surgery (VATS) lobectomy on the autonomic nervous system control of the heart, and to better guide future studies aimed at surgical risk stratification, we measured continuous HRV starting 2–4 days before surgery and ending 9 days after VATS in an Enhanced Recovery After Surgery (ERAS) setting, aimed at improving perioperative care and postoperative outcome [6, 7].

## Methods

### Patient recruitment and demographics

**Fig. 1:**
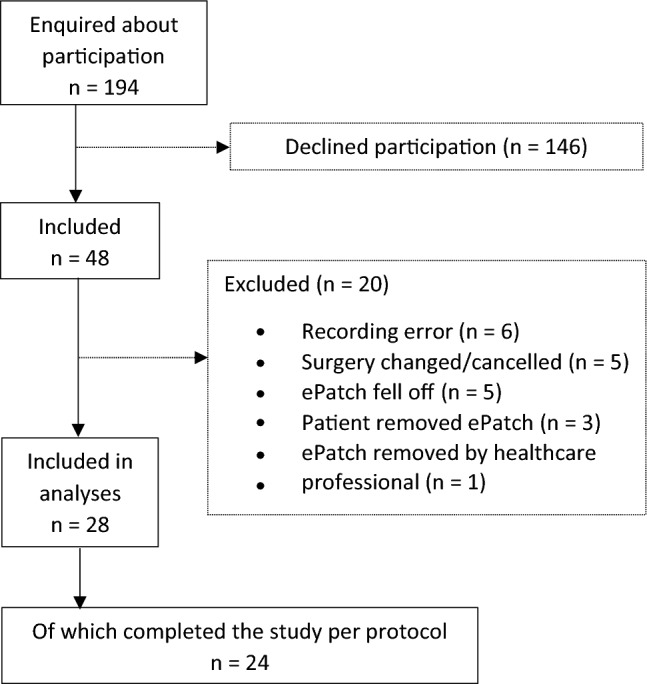
Patient flow with reasons for exclusion. Four patients had partially usable recordings and were therefore included in analyses

Patients were included in the study by a research nurse or a research fellow when they were referred to VATS lobectomy at the Department of Cardiothoracic Surgery at Rigshospitalet. Inclusion criteria were: Age ≥ 18 years and scheduled to undergo elective VATS lobectomy. The exclusion criteria were: Diabetes mellitus, known autonomic dysfunction, cardiac arrhythmias, prolonged postoperative stay in the intensive care unit, conversion to open thoracotomy, segmentectomy or wedge resection, and pacemaker treatment. 24 patients finished the study as per protocol (Fig. 1), 4 additional patients had substantial noise in part of their recordings, but still had some usable continuous data recordings (e.g., data from the preoperative period until the fourth postoperative day). These 4 patients were included in analyses when possible. Patient demographics and intraoperative data were gathered from electronic health records. One patient experienced a rash on the sternum after removing the ePatch, which persisted for a couple of weeks, but faded with no complications.

The need for ethical approval was waived by the local committee on health research ethics (case no. 20078836). Patients signed informed consent forms before participating in the study. Approval for data capture and storage was given by “Videncenter for Dataanmeldelser” (approval no. P-2021–25).

We did not perform a formal power calculation due to the exploratory hypothesis generating nature of the study.

### Perioperative care

Standard perioperative care included all components of the ERAS guidelines [7] and a standardized 3-port anterior technique [8]. Only a single chest drain is routinely used and connected to the digital drainage system Thopaz + (Medela, Switzerland) with a pressure of − 2 cm H_2_O [9]. For pain management, as described by Wildgaard et al. [10], the surgeon made a paravertebral single-shot block at 5 or more thoracic levels with a total of 20 ml bupivacaine 0.5% intraoperatively at the beginning of surgery. An intercostal catheter was inserted at the drain site using continuous bupivacaine 0.25% with a flow rate of 6 ml/h and remained there until chest drain removal. Additionally, the multimodal opioid-sparing regimen consisted of paracetamol, ibuprofen, and gabapentin. The criteria for chest drain removal and discharge have been described previously [11].

### HRV measurement

An ePatch 2.0 (Biotelemetry Inc., Sweden) was mounted on the skin below the jugular notch 2–4 days before surgery (PRE2, PRE4). The device recorded a one-channel ECG for 14 days with a recording frequency of 256 Hz. The patients wore the ePatch continuously for 12–14 days, until the ninth postoperative day (POD9). Some patients had the device removed during control x-ray, but had it reapplied shortly after. No recordings were lost due to removing the ePatch for x-rays. Patients were discouraged from submerging the ePatch in water, but otherwise had no physical restrictions due to participation in the study.

### Statistical analysis

HRV analysis was performed using Cardiscope ANALYTICS – Professional Edition (version 1.3.230) and Kubios HRV Premium (version 3.5.0), with no changes to the default settings, except for removing the automatic detrending of all indices by Kubios. HRV recordings were split into three 8-h periods per 24 h: NIGHT (11PM–7AM), DAY (7AM–3PM) and EVENING (3PM–11PM), and the entire periods were used for HRV analysis, without overlap between them. The NIGHT period was the first in a day, i.e., the NIGHT period of POD1 started at 11PM on the day the patient had surgery, the day period started at 7PM, and the EVENING period started at 3PM on POD1. The DAY recording on the day of surgery (DOS) was removed as it was a mixture of pre-, intra-, and postoperative values and as the patients would be under anesthesia for a part of it. All statistical analyses were performed in R (version 4.1.1) with R-studio (2022.02.3, Build 492) as the user interface. The data were checked for normality by visual inspection of Q-Q plots. We removed extreme outliers identified by the “rstatix package”. Continuous repeated measures data were automatically tested for sphericity by Mauchly’s test and underwent automatic Greenhouse–Geisser sphericity correction if they violated the sphericity assumption. ANOVA was performed if the assumptions were fulfilled, with post hoc pairwise t-tests. If there were complete data for a given parameter, the t-tests were paired. If the assumptions were violated, we used Friedman’s test, and post hoc paired Wilcoxon sign test if complete data was available. For discrete repeated measures data, we used Cochran’s Q test and subsequent McNemar pairwise test. We did not impute missing values and rows with missing data were excluded by the statistical software. As this was a hypothesis generating study, we decided to perform post hoc tests regardless of the ANOVA results but report only adjusted values. P-value adjustment was done by the “Hochberg” method. Significance level was set at *p* < 0.05. In some figures, we only present data at a higher significance level to improve the visual quality, otherwise detailed in the figure legend.

## Results

Table 1 presents patient demographics and perioperative characteristics. Median length of stay was 4 days.

**Table 1:** Perioperative characteristics

Pre- and perioperative characteristics	N = 28
Sex	
Female	13 (46%)
Male	15 (54%)
Age/years	67 ± (10)
BMI	25.6 ± (4.6)
ASA	
1	1 (3.6%)
2	5 (18%)
3	22 (79%)
Procedure length/min	90 [74–114]
Bleeding/ml	14 [5–42]
LOS/days	4 [2–9]

Data presented as: n (%); Mean ± (SD); Median [IQR]

### Heart rate variability

With reference to our previous publication [1], we chose to report data on SDNN, total power (TP), low-frequency divided by high-frequency power (LF/HF) and detrended fluctuation analysis alpha-1 (DFA1), as we identified these 4 indices to be associated with and of possible value in preoperative risk prediction [1]. We present more indices in the supplementary material.

#### *Standard Deviation of Normal-to-Normal beats*—*SDNN*

**Fig. 2:**
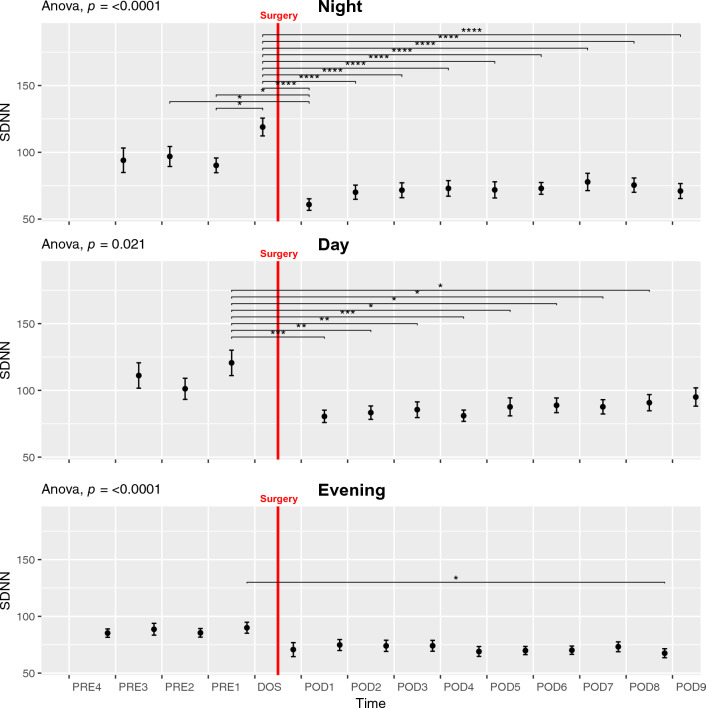
Course of Standard deviation of normal-to-normal beats (SDNN) in the perioperative period during the night, day, and evening. *p < 0.05, **p < 0.01, ***p < 0.001, **** *p* < 0.0001. One-way ANOVA followed by post-hoc paired t-test between time points adjusted for mass significance. Data presented as mean (dot) ± SE (error bars)

The variation in SDNN across the study was significant in NIGHT, DAY, and EVENING (ANOVA *p* < 0.03; Fig. 2) showing a peak in the DAY and EVENING periods on PRE1 and in the NIGHT before surgery. Pairwise t-test showed significant decreases from the preoperative to postoperative values, most prominently in the NIGHT where all postoperative values were significantly reduced from the DOS NIGHT (Fig. 2 Night, *p* < 0.0001). The DAY period showed similar results with significant reductions from the peak on PRE1 to POD1-8 (Fig. 2 Day, *p* < 0.05). In the EVENING period we only saw a significant reduction from the peak on PRE1 to POD8. SDNN showed a marked circadian variation (ANOVA *p* < 0.0001, Fig. 6A) with a peak during DAY compared to other periods and a significant difference between PRE1 DAY, EVENING and DOS NIGHT (*p* < 0.01, Fig. 6A). SDNN also exhibited postoperative circadian variation (ANOVA p < 0.0001, Supp. Figure 13A), with the highest numerical values on the DAY periods followed by EVENING, then NIGHT, in the following days, there was a shift to mimic the preoperative values on POD4. There were no significant differences between any individual days in the postoperative period.

#### *Total Power*—*TP*

**Fig. 3:**
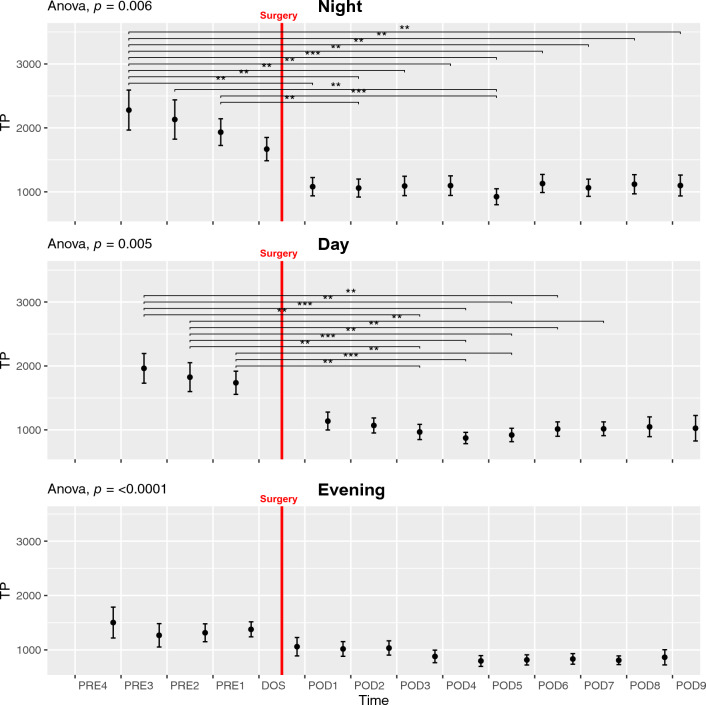
Course of Total Power (TP) in the perioperative period during night, day, and evening periods. One-way ANOVA fol lowed by post-hoc paired t-test between time points adjusted for mass significance. **p < 0.01, ***p < 0.001. To improve readability of the graph, only *p* < 0.01 are shown in the night and day periods. Subjects 9, 35 and 36 were removed from TP analysis due to being an extreme outlier. Data presented as mean (dot) ± SE (error bars)

We found a decline in TP throughout the preoperative phase with a further drop after surgery in NIGHT, DAY, and EVENING (ANOVA *p* < 0.007, Fig. 3). Pairwise t-tests revealed that all preoperative (PRE3-1) values were significantly higher than all postoperative values (POD1-8) in the NIGHT period. In the DAY period the same pattern emerged, except PRE1 to POD1 being insignificant (*p* = 0.08). We only demonstrated significant results with *p* < 0.001 in the NIGHT and *p* < 0.01 in the DAY. We did not find any statistically significant differences in the EVENING period in the pairwise comparison after adjusting for mass significance. (Fig. 3 Evening). TP also exhibited circadian variation with a peak in the nighttime (ANOVA *p* < 0.0001), but pairwise t-tests did not reveal any significant differences between individual time points after adjusting for mass significance (Fig. 6B). In the postoperative period it seems that, visually, there was a loss of circadian variation, that was reestablished on POD3 or POD4, with higher numerical values in the NIGHT, followed by DAY and then EVENING, but both ANOVA and paired t-tests were not statistically significant (Supp. Figure 13B).

#### *Low-Frequency divided by High-Frequency*—*LF/HF*

**Fig. 4:**
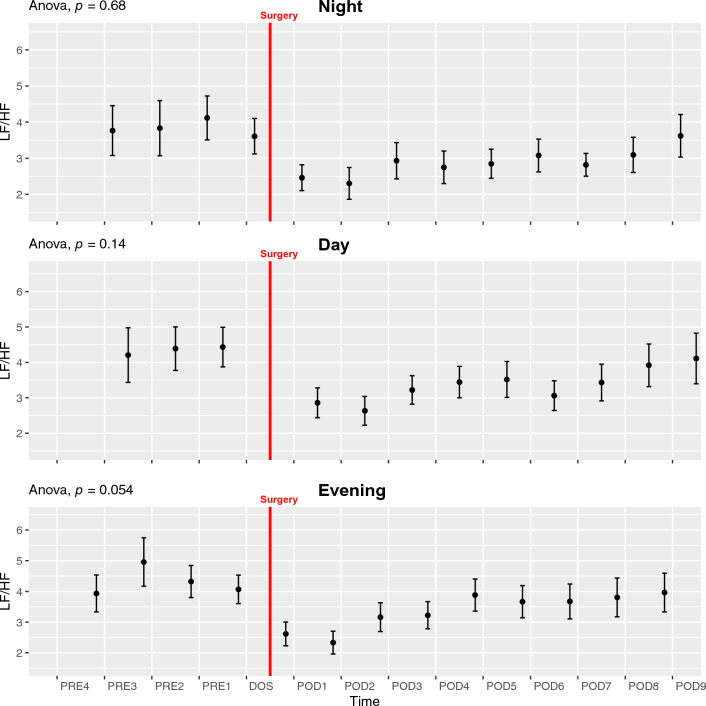
Course of low-frequency power divided by high-frequency power (LF/HF) in the perioperative period during night, day, and evening periods. One-way ANOVA showing significant fluctuations in evening period. Subjects 15 and 16 were removed from LF/HF analysis due to being extreme outliers. Data presented as mean (dot) ± SE (error bars)

We found no significant perioperative changes in LF/HF, although visually there seems to be a postoperative drop, followed by an increase (ANOVA *p* = 0.68, 0.14, and 0.054 for NIGHT, DAY, and EVENING respectively; Fig. 4). There was no was significant circadian variation in the preoperative period (ANOVA *p* = 0.23; Fig. 6C), but in the postoperative period there was a significant increase throughout the whole period, but without circadian variation (ANOVA *p* = 0.00034, Supp. Figure 14A).

#### *Detrended Fluctuation Analysis alpha-1*—*DFA1*

**Fig. 5:**
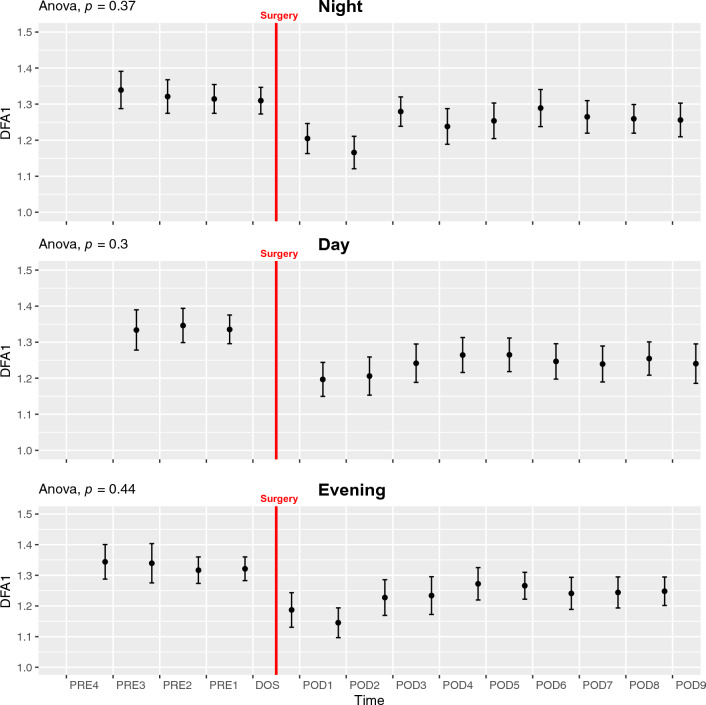
Course of detrended fluctuation analysis alpha-1 (DFA1) in the perioperative period during night, day, and evening periods. One-way ANOVA showing s ignificant fluctuations in NIGHT and EVENING periods. Data presented as mean (dot) ± SE (error bars)

**Fig. 6:**
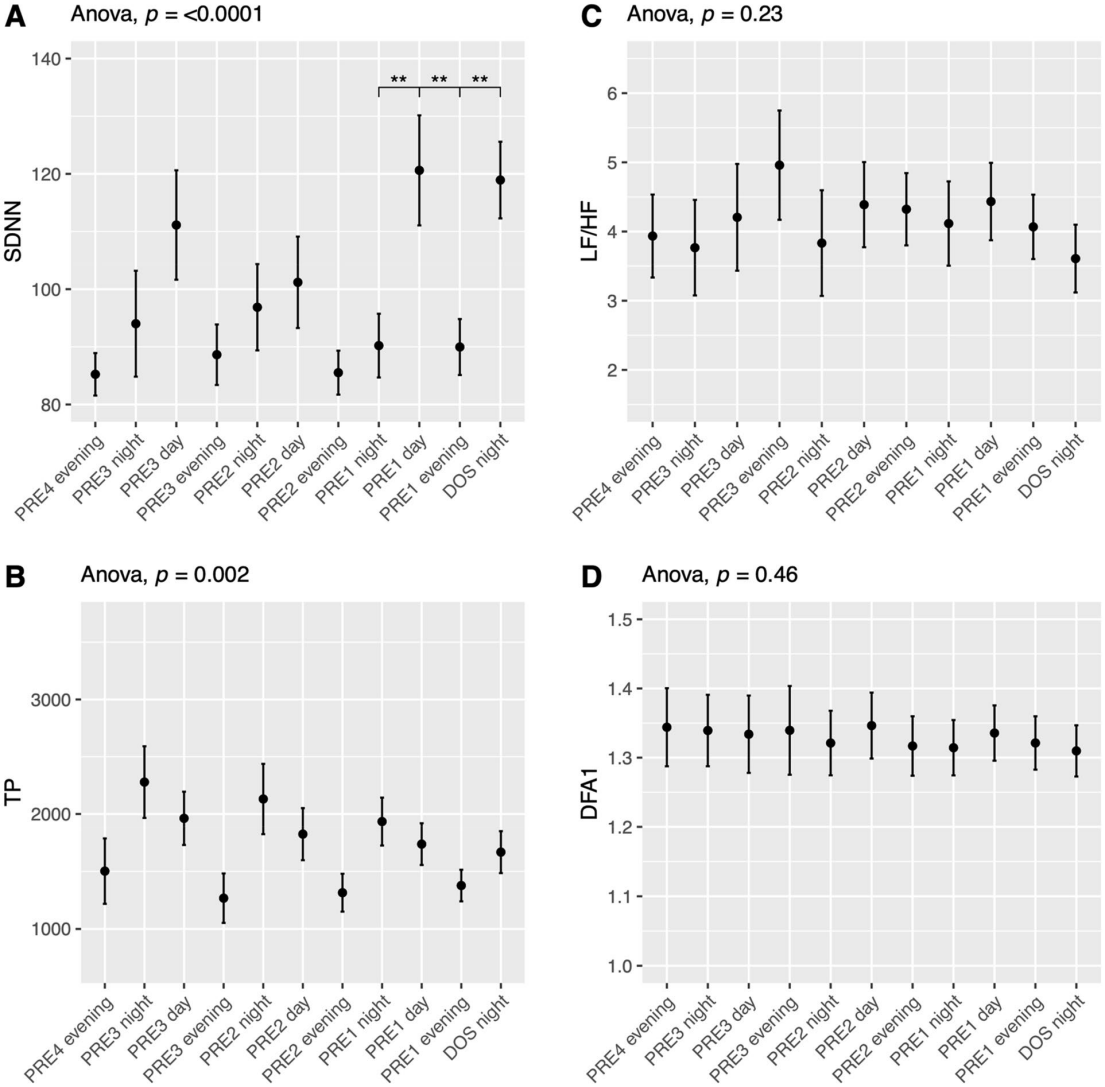
Preoperative time course of **A** SDNN showing significant circadian variation with increases from EVENING to NIGHT to DAY periods, and then a drop to EVENING again, **B** TP showing significant circadian variation, **C** LF/HF and D) DFA1 with no significant development during the preoperative period. Subjects 9, 35 and 26 were removed from TP analysis and subjects 14 and 16 were removed from LF/HF analysis because they were extreme outliers. Data presented as mean (dot) ± SE (error bars), **p < 0.01

There was no significant alterations in DFA1 across the study in any time periods (ANOVA *p* > 0.3, Fig. 5), nor did we see perioperative circadian variation (ANOVA *p* > 0.42, Fig. 6D, Supp. Figure 14B).

## Discussion

In this first detailed ERAS VATS study, we have shown that the perioperative time-course of several HRV indices depends on both what time of day they are measured, and on which day they are measured in relation to surgery. We found that indices measuring overall variability of the heart rate (SDNN and TP) were markedly reduced after VATS lobectomy, especially in the day- and nighttime, showing only slight signs of returning towards baseline at the end of the study 9 days after surgery. Additionally, it was shown that the same indices exhibit changes or loss of circadian variation in the early postoperative period, with a return of circadian variation 3–5 days following surgery. This cohort seemed to be a representative sample of patients treated at our institution, as they were similar to a previous larger cohort [12].

Although air leak is the primary reason for a prolonged hospital stay after VATS, other factors such as pain, pneumonia, and postoperative atrial fibrillation also contributes to longer hospitalization periods [11, 12]. Preoperative HRV has been shown to be associated with these complications in various surgical settings, including thoracic surgery [1, 13]. Furthermore, HRV has been linked to varying molecular inflammatory response trajectories following abdominal surgery, with patients exhibiting lower short-term variation showing higher levels of C-reactive protein [14]. This suggests a relationship between inflammation and changes in HRV.

No studies have examined the correlation between HRV and postoperative outcome in VATS, but the results presented in this study will aid in designing such future procedure-specific trials. From our study it seems that SDNN or TP in the night- or daytime would be the HRV measures most sensitive to the surgical insult and continuous measurement across several days would be recommended. However, if one is interested in a stable measure across both time of day, and day in relation to surgery, LF/HF or DFA1 might be a suitable measure as they show little pre- and even perioperative variation.

The strengths of this study are that patients were followed continuously for a longer duration both pre- and postoperatively than previous studies in VATS and that we used more HRV indices than previously reported. Limitations are the small sample size, not allowing any secondary analysis of risk stratification and the partially missing data in 4 patients. We did not control for time of surgery, and therefore had to discard the daytime recording on the day of surgery, and the study was performed in free-roaming subjects limiting comparison to shorter recordings under resting conditions.

A substantial number of included patients (n = 11) had problems related to mounting the device, either resulting in recording errors (loose connection of an electrode to the skin) or the device falling off. In a similar study in total hip arthroplasty we did not have these challenges to the same degree. Moving forward, special care should be taken that the device is properly mounted on the patient to ensure optimal data capture.

In conclusion, for the first time in ERAS VATS, we have shown that HRV indices exhibit different responses to surgery, depending on both the day of recording in relation to surgery and what time of the day the recording was made. Secondly, that some indices exhibit marked circadian variation in the preoperative period and a loss of circadian variation in the immediate postoperative period followed by a return later. Total HRV was markedly reduced following surgery, whereas single, more specific measures were more stable. Finally, we have shown that long-term perioperative HRV in free-roaming individuals is possible in patients undergoing VATS lobectomy, and that this is a valid platform for studying perioperative HRV.

These results are valuable in designing future HRV studies in VATS aimed at preoperative risk stratification.

## Supplementary Information

Below is the link to the electronic supplementary material.

Supplementary file1 (DOCX 17307 KB)

## Data Availability

Anonymized datasets and scripts used for analysis can be obtained on reasonable request to the corresponding author.
