# Supplementary material for: Continuous perioperative heart rate variability monitoring in video-assisted thoracoscopic surgery lobectomy—a pilot study
Source: J Clin Monit Comput. 2023 May 27;37(4):1071–9. doi: 10.1007/s10877-023-01016-2 (PMC10372135; doi:10.1007/s10877-023-01016-2)
Supplement: Supplementary file 1 — Supplementary file1 (DOCX 17307 KB) [file 10877_2023_1016_MOESM1_ESM.docx]

Supplementary figures

Continuous perioperative heart rate variability monitoring in video-assisted thoracoscopic surgery lobectomy – a pilot study

**Supplementary figure 1:** Course of Mean NN (ms between heart beats) in the perioperative period. There were no significant differences between individual timepoints,


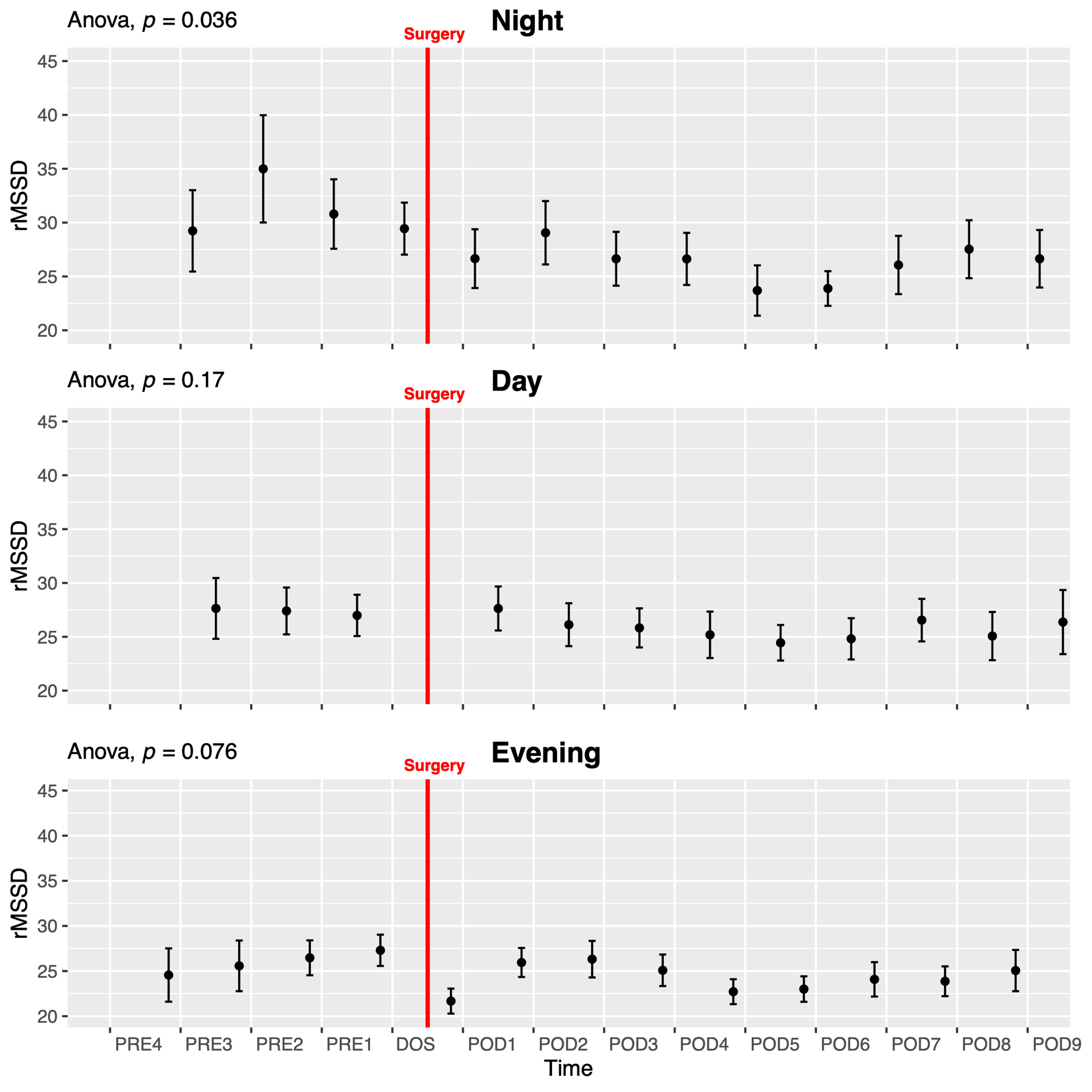


**Supplementary figure 2**: Course of the root mean square of the squared differences between adjacent NN intervals (rMSSD) in the perioperative period. One-way ANOVA showing higher values in the preoperative DAY period, followed by post-hoc pairwise t-test between timepoints adjusted for mass significance, with no significant results. Data presented as mean (dot) ± SE (error bars).


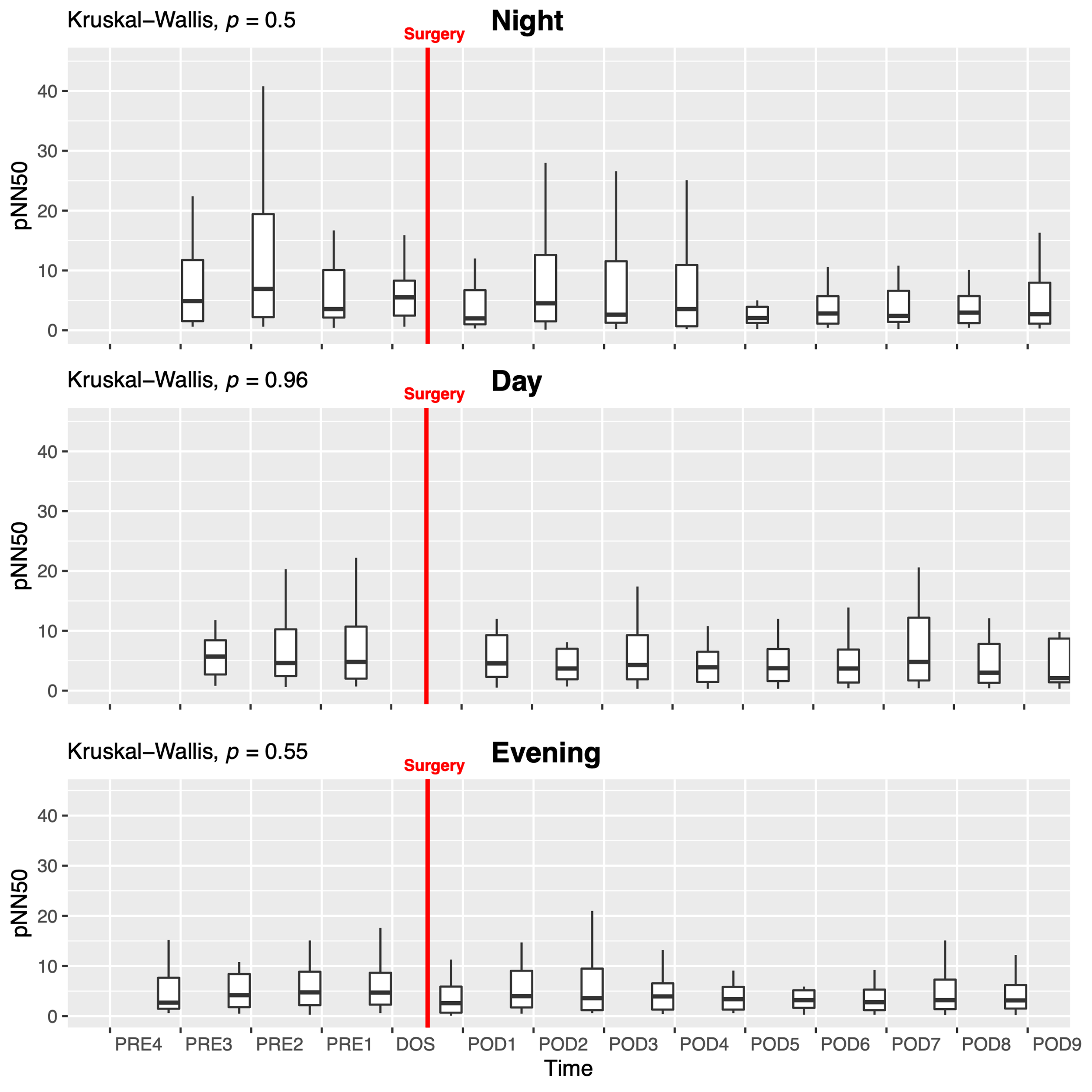


**Supplementary figure 3**: Course of percentage of NN intervals differing by more than 50 ms (pNN50) in the perioperative period. Due non-normality we performed Kruskal-Wallis test followed by post-hoc pairwise Wilcoxon test between timepoints, adjusted for mass significance and presented data as boxplots. Extreme outliers have been removed from the plot to improve visual presentation but is included in analysis. There were no significant results.


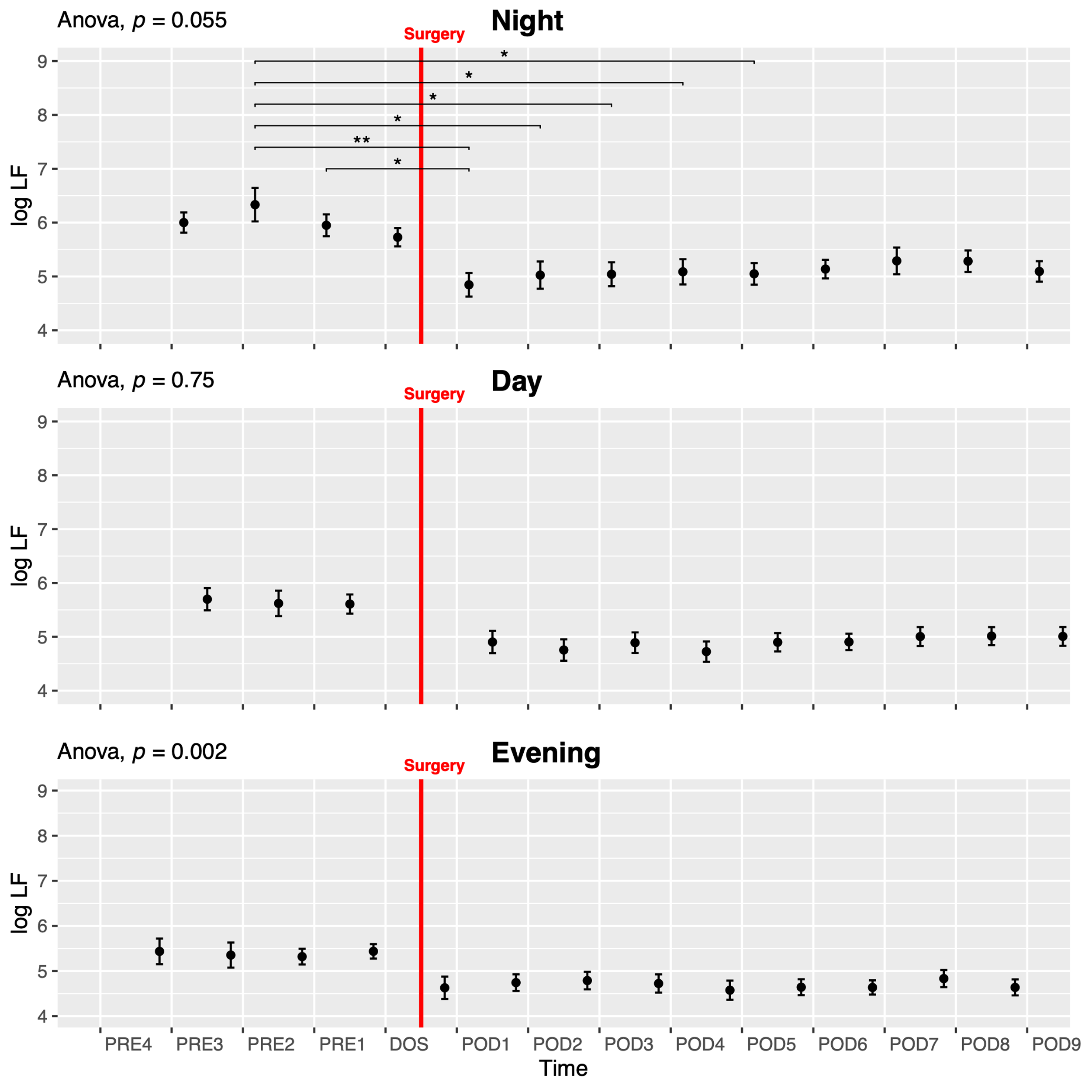


**Supplementary figure 4**: Course of the logarithm of low frequency power (log LF) in the perioperative period. One-way ANOVA followed by post-hoc pairwise t-test between timepoints adjusted for mass significance, with significantly higher values at multiple preoperative NIGHT periods compared to the postoperative NIGHT results. Subject 36 was removed from analysis due to being an extreme outlier. Data presented as mean (dot) ± SE (error bars).


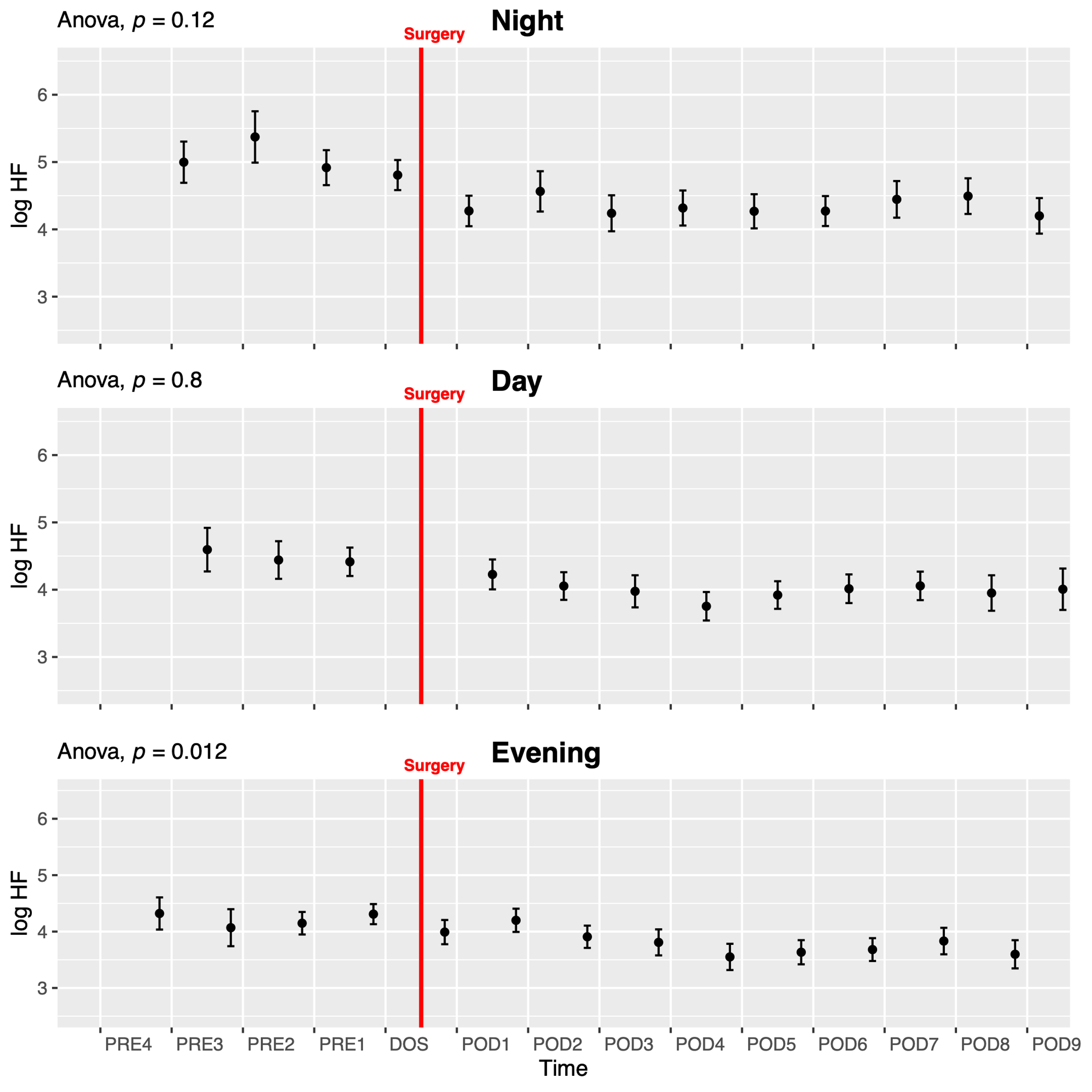


**Supplementary figure 5**: Course of the logarithm of high frequency power (log LF) in the perioperative period. One-way ANOVA showing a significant drop across the study period in the EVE periods, followed by post-hoc pairwise t-test between timepoints adjusted for mass significance. Data presented as mean (dot) ± SE (error bars).


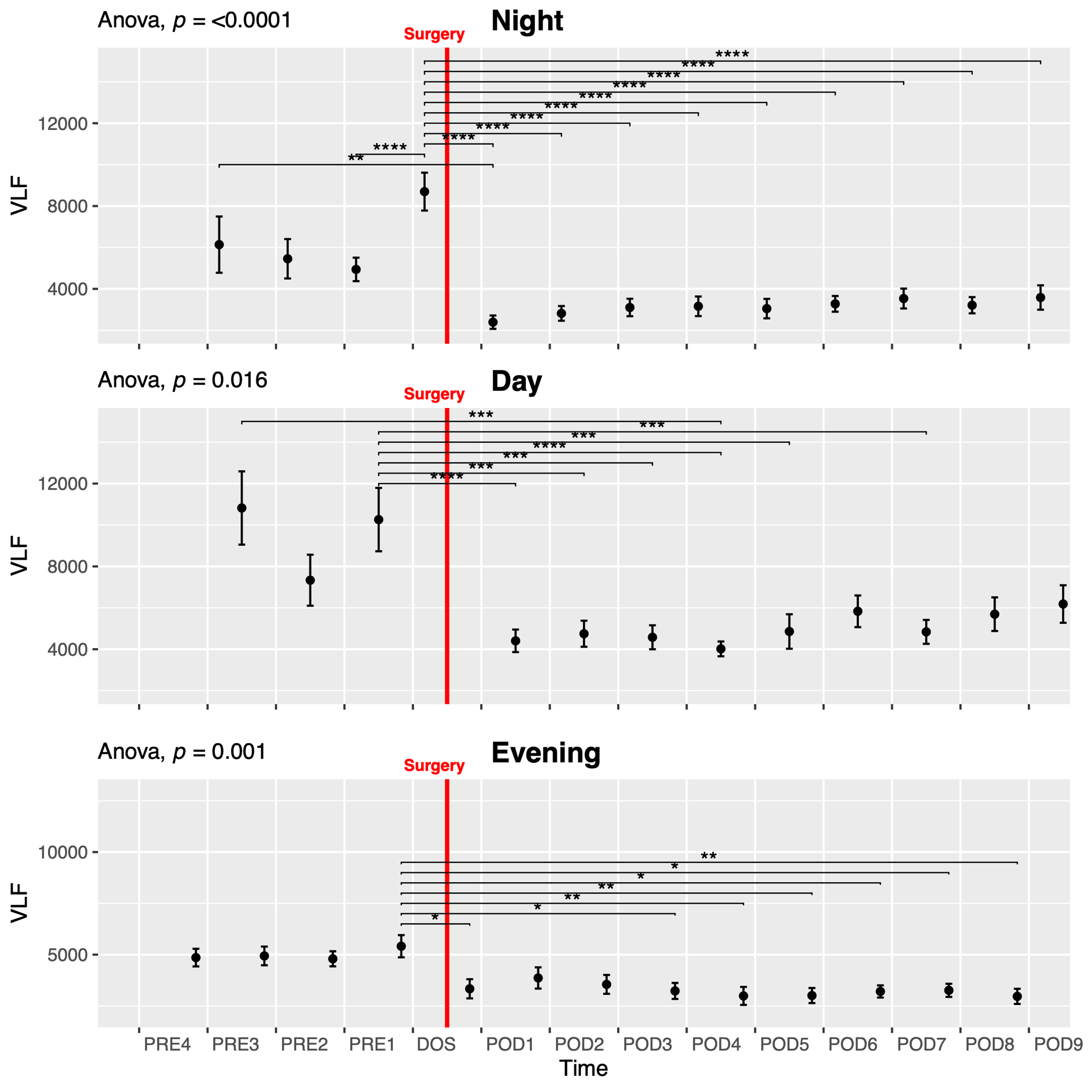


**Supplementary figure 6**: Course of very low frequency power (VLF) in the perioperative period. One-way ANOVA followed by post-hoc pairwise t-test between timepoints adjusted for mass significance, showing significant reductions in the postoperative period. Subjects 9 and 35 were removed from analysis due to being extreme outliers. Only p values <0.01 are shown in the NIGHT graph and values <0.001 are shown in the DAY graph. *p<0.05, **p<0.01, ***p<0.001, ****p<0.0001. Data presented as mean (dot) ± SE (error bars).


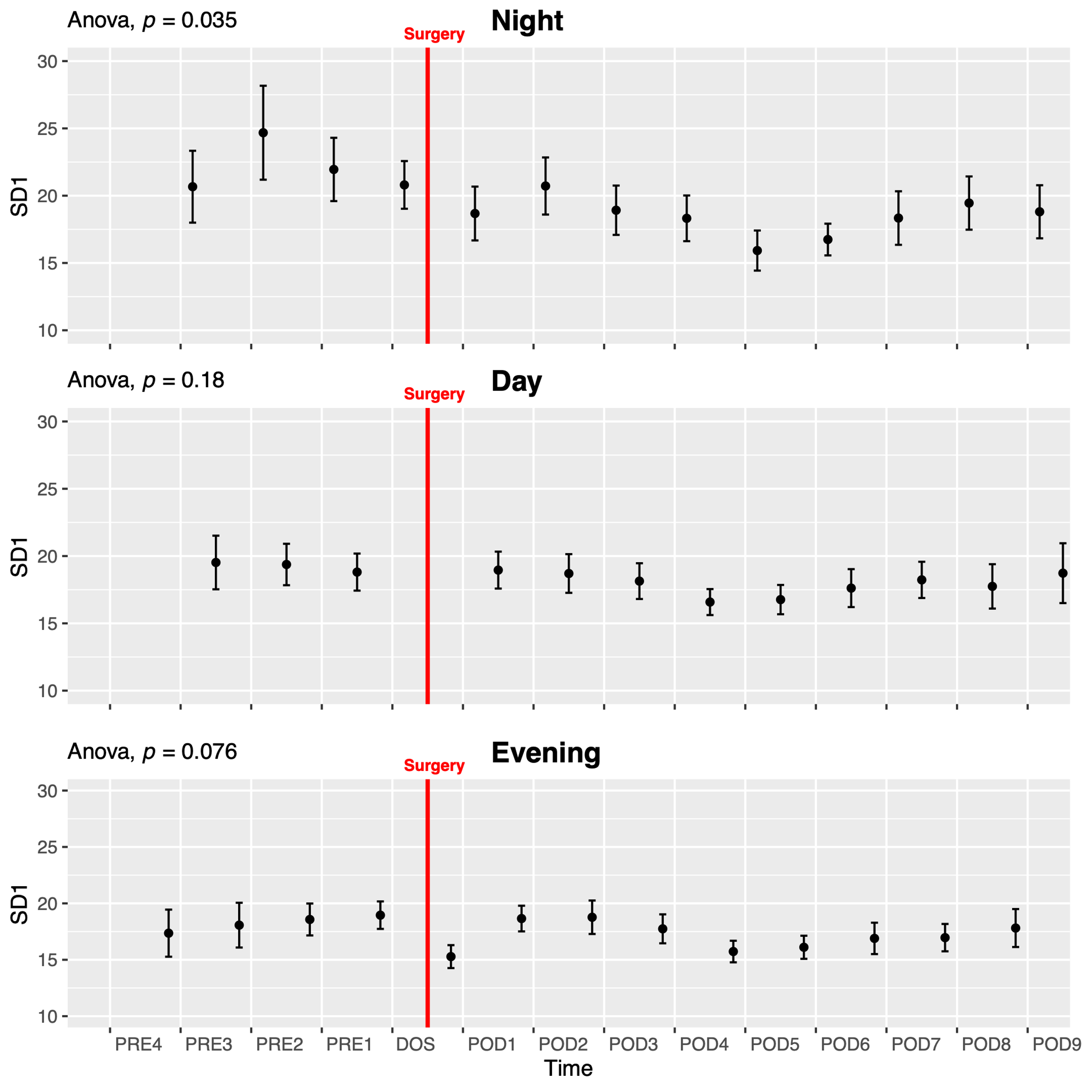


**Supplementary figure 7**: Course of nonlinear SD1 in the perioperative period. One-way ANOVA, showing a significant drop across the study period in the DAY periods and a possible drop after surgery in the EVE period. Post-hoc pairwise t-test between timepoints adjusted for mass significance did not reveal any significant differences between individual timepoints. Subject 16 removed from analysis due to being an extreme outlier. Data presented as mean (dot) ± SE (error bars).


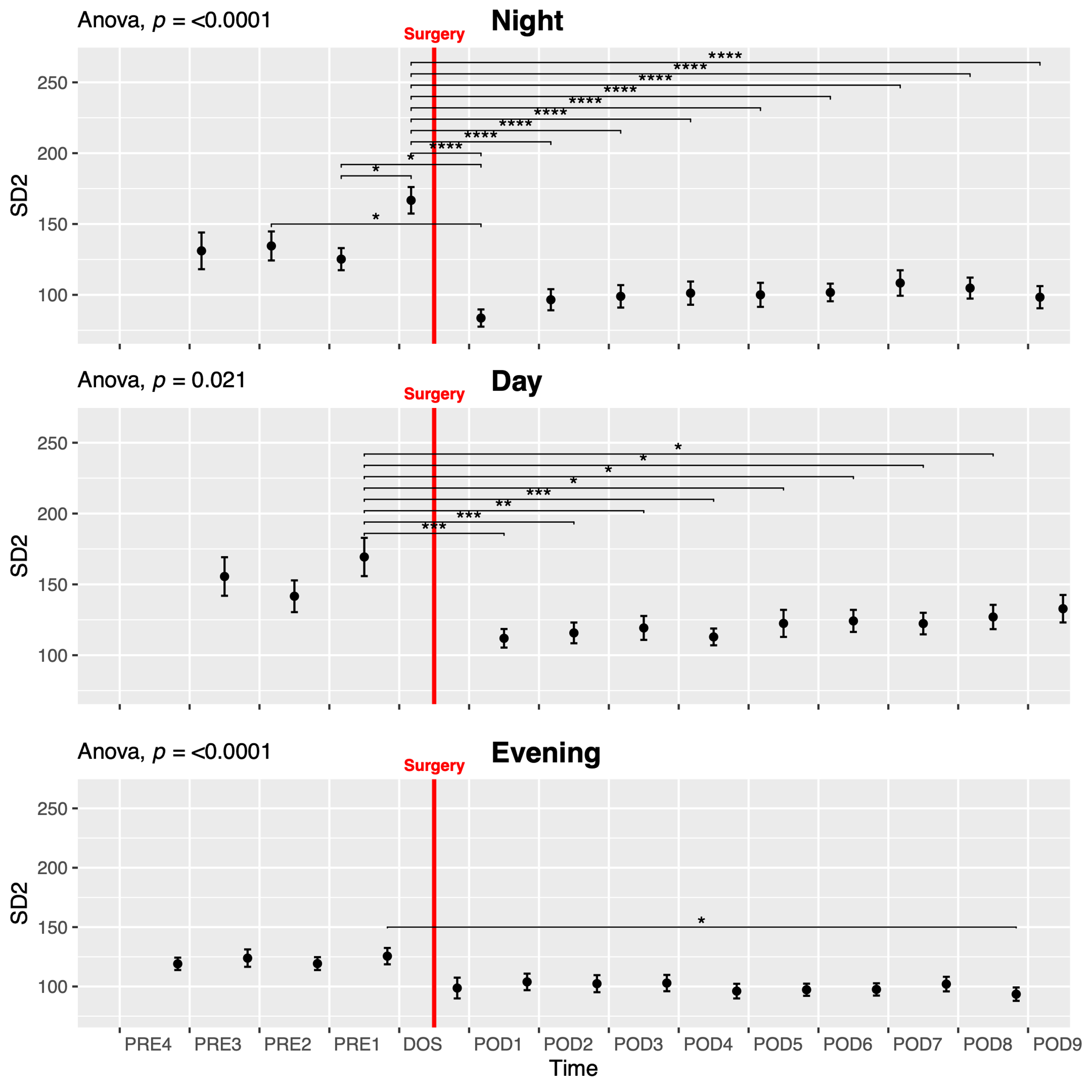


**Supplementary figure 8**: Course of nonlinear SD2 in the perioperative period. One-way ANOVA followed by post-hoc pairwise t-test between timepoints adjusted for mass significance, showing significant reductions following surgery, particularly in the NIGHT and DAY times. **p*<0.05, ***p*<0.01, ****p*<0.001, *****p*<0.0001. Data presented as mean (dot) ± SE (error bars).


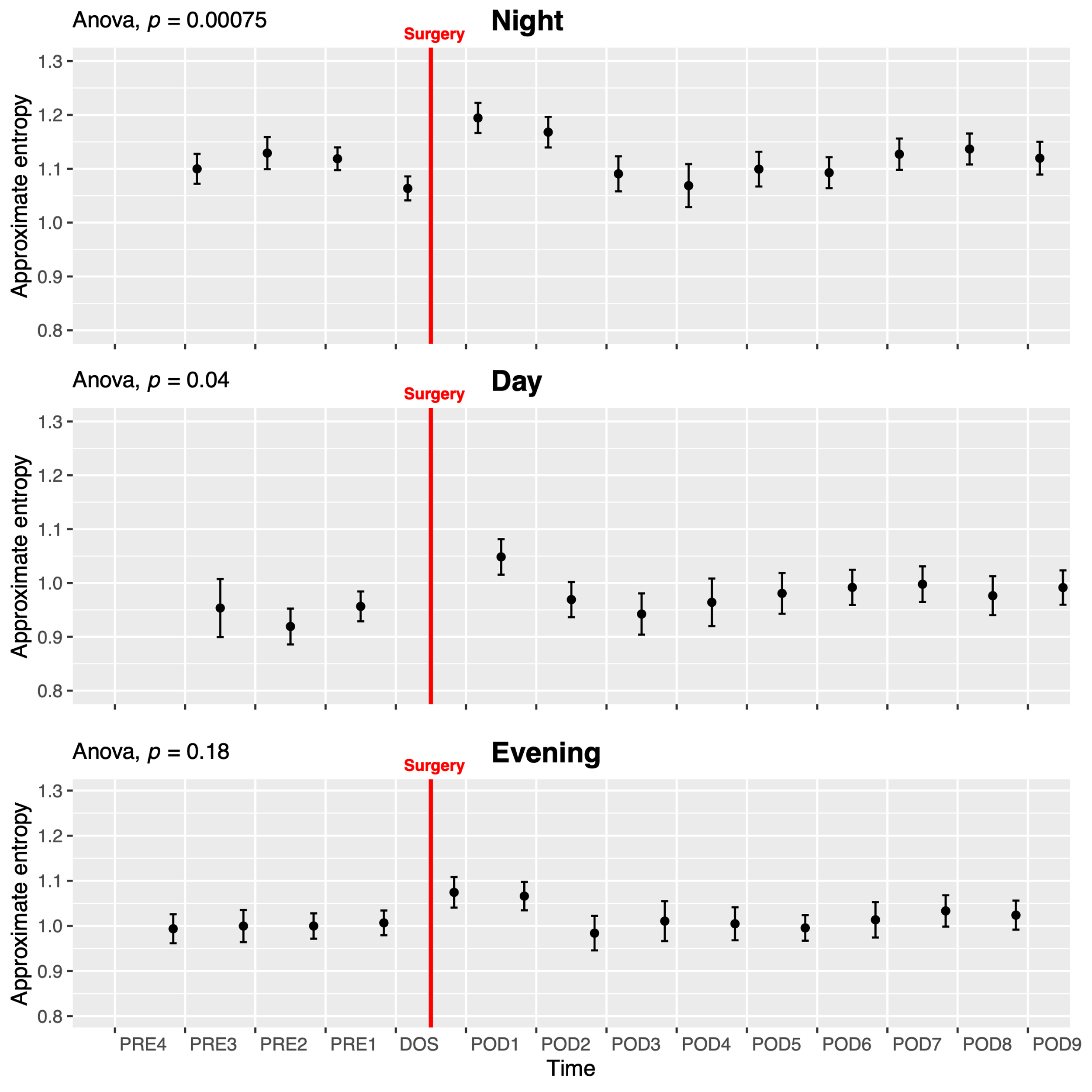


**Supplementary figure 9**: Course of approximate entropy in the perioperative period. One-way ANOVA showing increased entropy following surgery in the NIGHT and DAY periods. Post-hoc pairwise t-test between timepoints, adjusted for mass significance, did not reveal any significant differences. Data presented as mean (dot) ± SE (error bars).


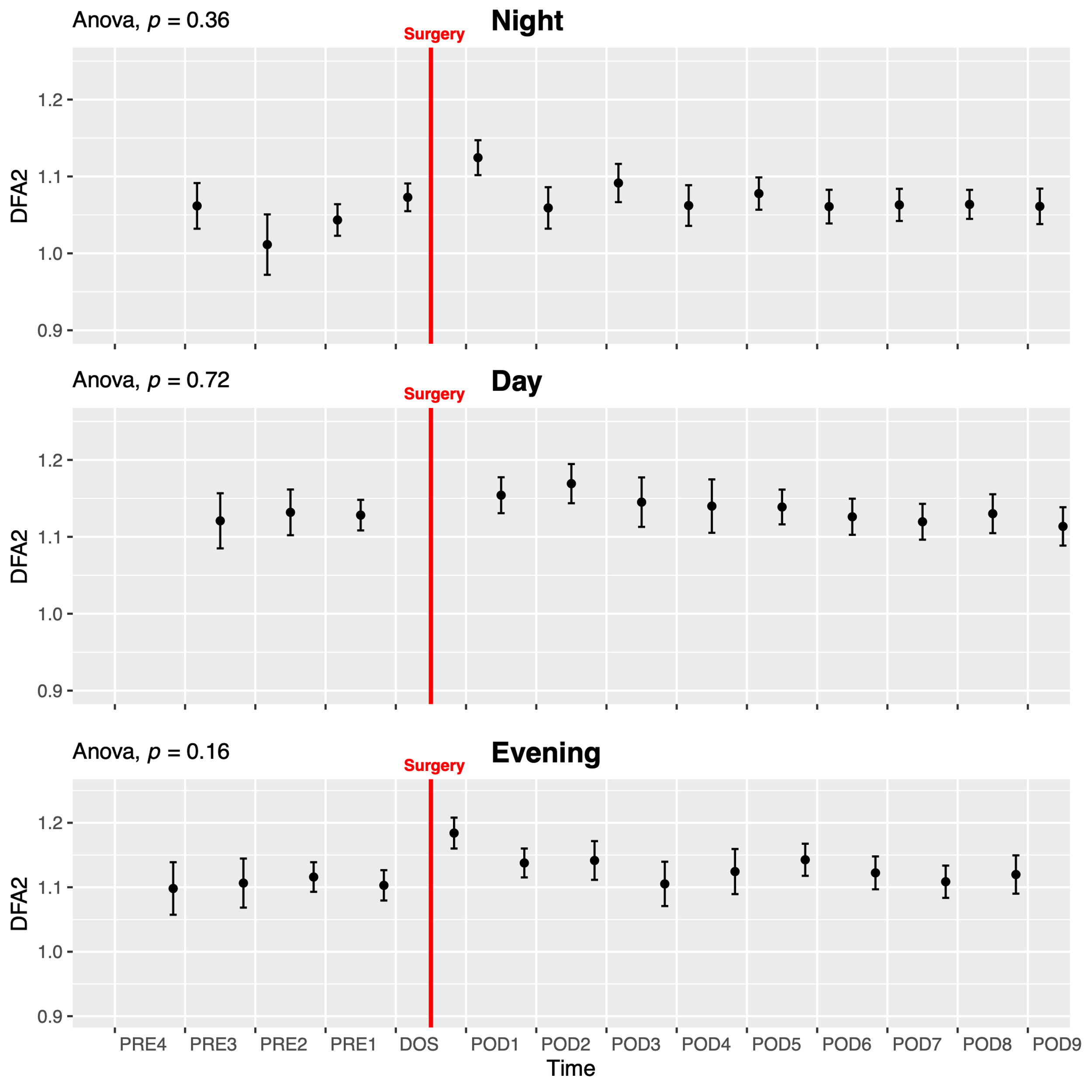


**Supplementary figure 10**: Course of detrended fluctuations analysis α2 (DFA2) in the perioperative period. One-way ANOVA followed by post-hoc pairwise t-test between timepoints adjusted for mass significance. Data presented as mean (dot) ± SE (error bars).


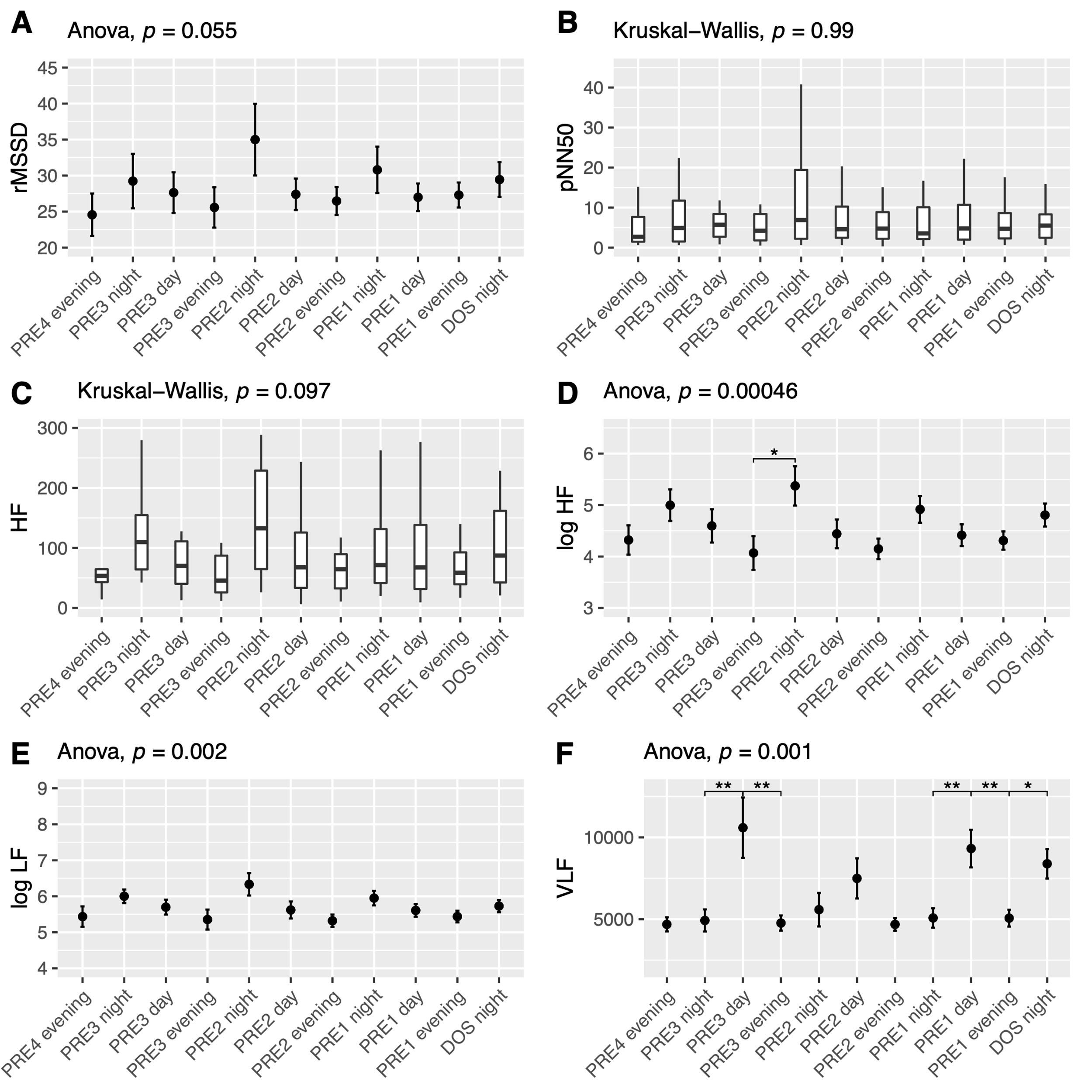


**Supplementary figure 11**: Preoperative time course of A) rMSSD showing near-significant circadian variation in ANOVA with values in the NIGHT periods, B) pNN50 showing no preoperative circadian variation, C) HF showing a trend towards higher values in the NIGHT periods, D) Log HF exhibiting circadian variations in ANOVA higher values at night, confirmed by post-hoc pairwise t-test on the PRE2 NIGHT period, E) Log LF showing circadian variation in ANOVA with a drop throughout the day, F) VLF showing higher values in the DAY periods in and on the night before surgery. Subjects 9 and 35 were removed from VLF analysis and subject 36 was removed from log LF analysis, all due to being extreme outliers. Data presented as mean (dot) ± SE (error bars). **p*<0.05, ***p*<0.01.


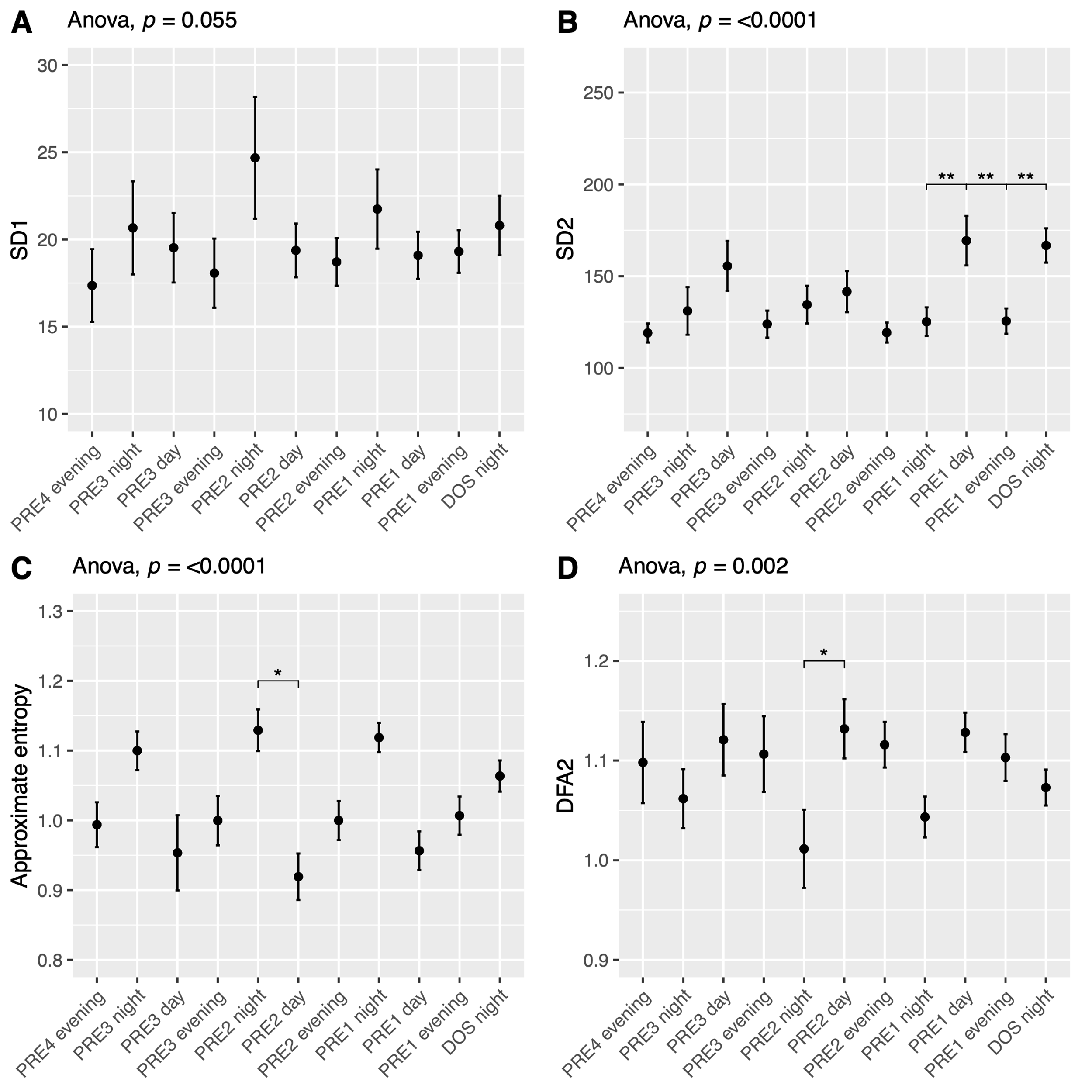


**Supplementary figure 12**: Preoperative time course of A) SD1 showing a trend towards a decrease throughout the day in ANOVA, B) SD2 showing increase in values towards the DAY periods in ANOVA and significantly higher values the DAY and NIGHT before surgery, compared to adjacent periods, C) Approximate entropy showing higher values in the NIGHT compared to DAY periods in ANOVA, confirmed by post-hoc pairwise test on PRE2 night, and D) DFA2 showing lower NIGHT period values in ANOVA as well as on PRE2 NIGHT in pairwise t-test. Subject 16 was removed from SD1 analysis due to being an extreme outlier. Data presented as mean (dot) ± SE (error bars). **p*<0.05, ***p*<0.01.

**Supplementary figure 13**: Preoperative time course of A) SD1 showing a trend towards a decrease throughout the day in ANOVA, B) SD2 showing increase in values towards the DAY periods in ANOVA and significantly higher values the DAY and NIGHT before surgery, compared to adjacent periods, C) Approximate entropy showing higher values in the NIGHT compared to DAY periods in ANOVA, confirmed by post-hoc pairwise test on PRE2 night, and D) DFA2 showing lower NIGHT period values in ANOVA as well as on PRE2 NIGHT in pairwise t-test. Subject 16 was removed from SD1 analysis due to being an extreme outlier. Data presented as mean (dot) ± SE (error bars). **p*<0.05, ***p*<0.01.

**Supplementary figure 14**: Preoperative time course of A) SD1 showing a trend towards a decrease throughout the day in ANOVA, B) SD2 showing increase in values towards the DAY periods in ANOVA and significantly higher values the DAY and NIGHT before surgery, compared to adjacent periods, C) Approximate entropy showing higher values in the NIGHT compared to DAY periods in ANOVA, confirmed by post-hoc pairwise test on PRE2 night, and D) DFA2 showing lower NIGHT period values in ANOVA as well as on PRE2 NIGHT in pairwise t-test. Subject 16 was removed from SD1 analysis due to being an extreme outlier. Data presented as mean (dot) ± SE (error bars). **p*<0.05, ***p*<0.01.

**Supplementary figure 15**: Preoperative time course of A) SD1 showing a trend towards a decrease throughout the day in ANOVA, B) SD2 showing increase in values towards the DAY periods in ANOVA and significantly higher values the DAY and NIGHT before surgery, compared to adjacent periods, C) Approximate entropy showing higher values in the NIGHT compared to DAY periods in ANOVA, confirmed by post-hoc pairwise test on PRE2 night, and D) DFA2 showing lower NIGHT period values in ANOVA as well as on PRE2 NIGHT in pairwise t-test. Subject 16 was removed from SD1 analysis due to being an extreme outlier. Data presented as mean (dot) ± SE (error bars). **p*<0.05, ***p*<0.01.

**Supplementary figure 16:** Preoperative time course of A) SD1 showing a trend towards a decrease throughout the day in ANOVA, B) SD2 showing increase in values towards the DAY periods in ANOVA and significantly higher values the DAY and NIGHT before surgery, compared to adjacent periods, C) Approximate entropy showing higher values in the NIGHT compared to DAY periods in ANOVA, confirmed by post-hoc pairwise test on PRE2 night, and D) DFA2 showing lower NIGHT period values in ANOVA as well as on PRE2 NIGHT in pairwise t-test. Subject 16 was removed from SD1 analysis due to being an extreme outlier. Data presented as mean (dot) ± SE (error bars). **p*<0.05, ***p*<0.01.

**Supplementary figure 17**: Preoperative time course of A) SD1 showing a trend towards a decrease throughout the day in ANOVA, B) SD2 showing increase in values towards the DAY periods in ANOVA and significantly higher values the DAY and NIGHT before surgery, compared to adjacent periods, C) Approximate entropy showing higher values in the NIGHT compared to DAY periods in ANOVA, confirmed by post-hoc pairwise test on PRE2 night, and D) DFA2 showing lower NIGHT period values in ANOVA as well as on PRE2 NIGHT in pairwise t-test. Subject 16 was removed from SD1 analysis due to being an extreme outlier. Data presented as mean (dot) ± SE (error bars). **p*<0.05, ***p*<0.01.
